# Bridging the Wound Gap: Interim Results From Randomized Trials Evaluating Dehydrated Human Amnion-Intermediate Layer-Chorion Membrane for the Treatment of Non-healing Diabetic Foot Ulcers

**DOI:** 10.7759/cureus.97875

**Published:** 2025-11-26

**Authors:** Hunter J Hall, Ann Keenan, Kurt Massey, Walter F D'Costa, Peter Moyer

**Affiliations:** 1 Medical School, The University of Toledo College of Medicine and Life Sciences, Toledo, USA; 2 Podiatry, Purvis Foot and Ankle Center, Rocky Mount, USA; 3 Advanced Foot and Ankle Center, Piedmont HealthCare, Mooresville, USA; 4 Podiatry, Northern California Foot and Ankle Center of Santa Rosa, Santa Rosa, USA

**Keywords:** allograft, chronic wound care, diabetes mellitus, diabetic chronic wounds, diabetic foot ulcer (dfu), human placental tissue, skin substitute

## Abstract

Introduction

Patients with diabetes frequently develop diabetic foot ulcers, a significant clinical pathology that results in increased morbidity and mortality. Standard of care (SOC) treatment has been insufficient to prevent the poor outcomes associated with these wounds. This analysis aims to evaluate the supplementation of SOC with a human tissue allograft.

Methods

This evaluation is of two prospective, multicenter, randomized clinical studies (ELITE - ongoing and CAMPLIFE - terminated) comparing a dehydrated human amnion-intermediate layer-chorion membrane (dHAICM) tissue allograft (AmchoPlast^®^, Cellution Biologics, Inc., Roswell, GA, US) as a supplement to SOC (dHAICM + SOC) versus SOC alone. Eligible target ulcers must have been present for a minimum of four weeks with SOC treatment and not have reduced in size by more than 20% during the screening period. Participants were treated weekly for up to 10 weeks in the ELITE study and 12 weeks in the CAMPLIFE study.

Results

Across the ELITE and CAMPLIFE studies, 90 participants were screened, with 64 total participants remaining eligible at the randomization visit who subsequently were enrolled. Of those, a total of 53 participants were eligible for inclusion in this analysis, 29 of whom had completed the study per protocol as of the data cutoff time for this analysis. A total of 18 participants (34%) achieved complete wound closure of their ulcers. In the SOC alone group, four participants healed (17%) compared to 14 (50%) in the dHAICM + SOC group, a statistically significant difference in healing between the two groups (P = 0.019). Utilizing a non-pooled sample from the ELITE study was also statistically significant; 12 participants (71%) in the dHAICM + SOC group healed, compared to three participants (23%) in the SOC alone group (P = 0.0253). A reduction in wound area of 71% ± 18.2% was observed for the dHAICM + SOC group and 46% ± 16.9% for the SOC alone group; however, this reduction did not reach statistical significance (P = 0.0517). In the interim analysis of the data from the ELITE study, a wound area reduction of 78% ± 23.3% was achieved by the dHAICM + SOC vs. a 38% ± 20.1% reduction for SOC alone, a statistically significant difference between the two groups (P = 0.018). No statistically significant improvements in pain scores, Wound Quality of Life assessments, or Forgotten Wound Score assessments were observed in either the pooled sample or the ELITE only analysis.

Discussion

Both analyzed study populations showed better outcomes for wound closure and wound area reduction when SOC was supplemented with dHAICM, although wound area reduction only reached statistical significance for the ELITE interim analysis.

Conclusions

Supplementing SOC treatment for diabetic foot ulcers increases the likelihood of the ulcer healing completely and improves the reduction in wound area.

## Introduction

Diabetes continues to be a prevalent chronic condition associated with an increased risk for numerous adverse sequelae. Diabetes is a leading cause of cardiovascular disease, contributing significantly to morbidity and mortality [1]. In 2017, the number of type 2 diabetes-afflicted individuals was approximately 462 million, or about 6.3% of the global population [2]. Globally, in 2024, approximately 580 million adults aged 20-79 were affected by type 2 diabetes, 11.1% of this population, and an increase of almost 120 million in seven years [1]. The prevalence of diabetes continues to increase in every region globally, consistently outpacing future projections, contributing to its role as a major global health problem [1]. The prevalence of type 2 diabetes is also increasing among adults under 40 and children [1]. Type 2 diabetes comes with a number of potential complications including vascular, renal, ophthalmic, hepatic, and neurological disorders that can cause significant health consequences, especially for racial and ethnic minorities [3,4].

A major complication resulting from type 2 diabetes includes chronic diabetic wounds. Chronic diabetic wounds are characterized by impaired healing, prolonged inflammation, and reduced epithelization kinetics [5]. Diabetic foot ulcers (DFUs) are a subtype of diabetic chronic wounds, usually the result of increased pressure on weight-bearing areas of the foot, friction from poorly fitting shoes, gait abnormalities, or sustained injuries [6]. One-third of diabetic patients are expected to develop a DFU in their lifetime [7]. Patients with cardiovascular disease, kidney disease, peripheral artery disease, peripheral neuropathy, or foot abnormalities are at higher risk for developing DFUs, along with patients who are inactive or smoke [6]. DFU incidence can be expected to increase with the increasing number of patients with type 2 diabetes.

DFUs are a pernicious wound type for diabetic patients with a five-year mortality rate reported at over 30.5% in the United States [8]. In a study of over 20,000 patients in the United Kingdom, DFUs were associated with an even higher five-year mortality rate of 42% [9]. DFUs precede 84% of diabetes associated lower limb amputations [5]. In the United States, over 507,000 Medicare beneficiaries were affected by DFUs in 2019, representing 0.8% of all Medicare beneficiaries [10]. Medicare beneficiaries being treated for DFUs represent over 1.3% of all people with diabetes in the United States and represent a significant use of provider resources in their management [1,10]. Treatment costs of DFUs for both private insurers and Medicare are estimated to be $9-$13 billion annually in the United States (~$4 billion from Medicare in 2019) [10,11]. This sum does not include the average $3,259 in work-loss costs per DFU patient compared to matched controls without DFUs [10,11]. Additionally, DFUs have a recurrence rate estimated to be greater than 60%, illustrating the need for effective treatment methodologies to alleviate this significant social and economic burden [7,12].

The established gold standard of care (SOC) for DFUs, which includes debridement, offloading, infection management, maintaining proper moisture balance, and revascularization (in patients with significant ischemia), is often insufficient to effectively treat DFUs [13]. Despite the significant financial and humanitarian disease burden represented by DFUs, as well as the lack of effective treatment modalities, there is a distinct gap in funding for research on DFUs. Only 0.17% of National Institutes of Health (NIH) diabetes research funding was allocated to DFU-related projects between 2002 and 2011 [14]. The cost of DFU treatment significantly outpaces the expenditure and growth of research funds.

One treatment modality for the treatment of DFUs gaining attention in recent years is the use of dehydrated human placental tissues (dHPTs) as allografts, which are donated human tissue intended for transplant into another human patient. These products provide several potential benefits, which include providing a protective environment for healing and structural support for soft tissue defects, aiding in epithelial cell migration, stimulating angiogenesis, reducing protease activity in the wound bed, improving functional performance, reducing scar formation, and allowing the tissue healing cascade to express itself [15,16]. The use of human placental tissue allografts as an adjunctive therapy to SOC has shown promise for the treatment of non-healing DFUs. Despite the increased cost associated with the allograft itself, these products could significantly reduce the cost to treat an individual patient with a DFU [16-20]. As a result of the significant ongoing costs of care for DFUs, any treatment modality that reduces the healing time can decrease the overall cost of DFU treatment. Previously published literature estimates a net financial benefit of approximately $5,000 per patient treated with dHPT over five years [21]. Although there has been recent interest in allografts for the treatment of DFUs, there is a lack of information on the efficacy of dehydrated human amnion-intermediate layer-chorion membrane (dHAICM) allografts in this indication, despite the clinical use of these allografts for the treatment of DFUs refractory to SOC treatment. Here, we present data from two prospective, randomized controlled studies evaluating a dHPT allograft (AmchoPlast^®^, Cellution Biologics, Inc., Roswell, GA, US) used as an adjunct therapy to SOC against SOC, supporting its use in DFU treatment through improving wound healing and quality of life while reducing pain scores.

## Materials and methods

Data source

Data were obtained from two prospective, multicenter, randomized clinical studies utilizing dHAICM tissue as a supplement to SOC (dHAICM + SOC) versus SOC alone, short-titled ELITE (NCT07014176) and CAMPLIFE (NCT06562296). IRB approval was obtained from Advarra’s IRB (IRB registration number 00000971) for both of the study protocols and all participant-facing materials, prior to any study conduct. Sample sizes for the ELITE and CAMPLIFE studies were determined based on having an 80% power to detect a 60% complete closure rate in the dHAICM + SOC group vs. 30% in SOC based on Fisher’s exact test at two-sided alpha = 0.05 with a 15% dropout rate, to ensure sufficient participants were enrolled. The sample size for ELITE will be adjusted based on this interim analysis.

Potential participants were screened for infection, past or current treatment with potentially interfering medication, vascular perfusion, and malnutrition. To be eligible for inclusion in either study, participants’ target ulcer must have been present for a minimum of four weeks with SOC treatment. During the screening period, potential participants were treated utilizing SOC, and any participants whose ulcer reduced in area by more than 20% were excluded from further participation. Participants who remained eligible were randomized to either SOC alone or dHAICM + SOC, at a ratio of 1:1, and were treated weekly for the duration of the study. SOC treatment included saline wound cleansing, sharp debridement, calcium alginate or foam dressing application, and offloading for plantar ulcers or those subject to external pressure. Prior to dressing with either calcium alginate or foam, dHAICM was applied to the wound, if applicable, based on randomization assignment. Both participants and study personnel were aware of the randomization assignment. The parameters of wound area, condition, Visual Analog Scale (VAS) pain assessment, Wound Quality of Life (WQoL), Forgotten Wound Score (FWS), and adverse events (AEs) were assessed throughout the treatment period. Wound area was measured utilizing a digital planimetry measurement device to ensure measurement consistency. ELITE participants were treated for up to 10 weeks, and CAMPLIFE participants were treated for up to 12 weeks. Participants whose wounds were fully healed prior to the end of the treatment period received one additional assessment visit to confirm if the ulcer remained healed.

ELITE study

The ELITE study has screened 49 participants and enrolled 35 participants across seven sites. An interim analysis of 30 enrolled participants who completed at least 50% of follow-up visits scheduled to occur prior to the scheduled data cutoff was included in the data presented here. This study continues to be open and enrolling.

CAMPLIFE study

The CAMPLIFE study screened 39 participants and enrolled 27 participants across four sites who met the eligibility criteria. Twenty-five participants presented with DFUs, and of those, 23 completed at least one follow-up visit. These participants missed no more than 50% of scheduled follow-up visits, making them eligible to be included in the analysis. Two participants were treated for venous leg ulcers and so were not included in the data presented here.

Study objectives and outcome measures

The primary objective of these studies was to determine the difference in the proportion of participants achieving complete wound closure by the study-specific endpoint (week 10 ELITE; week 12 CAMPLIFE) when treated with weekly dHAICM + SOC versus SOC alone in adults with DFUs refractory to four weeks of SOC therapy. For this interim analysis, confirmation of wound closure was assessed by the site principal investigator. The secondary objectives included determining the differences between ulcers treated with dHAICM + SOC versus SOC alone in the following: time to closure, percent area reduction (PAR), pain (VAS score), improvement in quality of life (WQoL and FWS questionnaires), and safety of dHAICM (frequency and nature of AEs).

Human tissue allograft product

dHAICM is a dehydrated human placental product derived from donated human tissue. This product is considered part of the broader category of dHPT allografts. The allograft product is a dehydrated multilayer placental tissue containing the amnion and chorion layers as well as the basement membrane and trophoblast. dHAICM is sterilized via gamma irradiation. The study product was screened and tested in accordance with the American Association of Tissue Banks (AATB) standards. The process of acquiring the tissue and manufacturing was performed as per FDA 21 CFR 1271 and AATB guidelines. The brand name for the dHAICM product utilized in these studies was AmchoPlast^®^ supplied by Cellution Biologics, Inc.

Statistical approach

Outcome measures were examined for the treatment groups (dHAICM + SOC vs. SOC) utilizing two-tailed Fisher’s exact test for calculation of P-values for categorical variables. Unpaired, two-tailed Student’s t-tests were utilized for P-value calculation of continuous variables. All evaluations were tested at a 95% confidence level for significance (P < 0.05 is considered statistically significant). Confidence intervals were constructed as a two-sided 95% confidence interval. For the calculation of endpoints, participant data from the most recently completed visit was utilized, if study participation was ongoing and final visit data were not yet available.

## Results

A total of 90 participants with DFUs entered the screening phase across the ELITE and CAMPLIFE studies, with 64 total participants remaining eligible at the randomization visit who subsequently enrolled. A total of 53 participants were eligible for inclusion in this analysis, having completed at least 50% of scheduled follow-up visits and at least one follow-up visit for subjects who withdrew early from the study. In this analysis, 17 participants from the ELITE study were randomized to dHAICM + SOC and 11 participants from the CAMPLIFE study, for a total of 28 dHAICM + SOC participants included in this analysis. ELITE randomized 13 participants to SOC alone and 12 participants from CAMPLIFE for a total of 25 participants treated with SOC alone included in this analysis. Two participants in the CAMPLIFE study randomized to dHAICM + SOC did not complete any follow-up visits after randomization and were not included in this analysis. Five participants from the ELITE study randomized to SOC alone and two participants randomized to dHAICM + SOC did not complete sufficient follow-up and were not included in this analysis.

Study population

The dHAICM + SOC and SOC alone groups exhibited similar clinical presentations at baseline, including size and duration of ulcer (Table 1), with no statistically significant differences in age, sex, race, BMI, HbA1c, ulcer duration, or ulcer size found between the two groups, in each study, as well as in the combined analysis.

**Table 1:** Demographics and ulcer characteristics at baseline visit Categorical data are represented as number of participants (%). Quantitative data are represented as mean (±standard deviation) or median (minimum, maximum). * indicates a statistically significant difference between groups at baseline (P < 0.05) based on two-tailed Fisher’s exact test. No quantitative data were statistically significantly different at baseline (P < 0.05) based on two-tailed, unpaired Student’s t-test. dHAICM: dehydrated human amnion-intermediate layer-chorion membrane; SOC: standard of care

	dHAICM + SOC; N = 28	SOC alone; N = 25
Age	60 (±12.1)	58 (±13.5)
Age ≥ 65 years	11 (39%)	9 (36%)
Sex: female	8 (29%)	13 (52%)
Sex: male	20 (71%)	12 (48%)
Race: Caucasian	18 (64%)	16 (64%)
Race: African American	6 (21%)	6 (24%)
Tobacco user	7 (25%)*	2 (8%)*
Ulcer location		
Plantar	18 (64%)	12 (48%)
Toe	5 (18%)	5 (20%)
Dorsal	0 (0%)*	6 (24%)*
Midfoot	1 (4%)	2 (8%)
Heel	3 (11%)	1 (4%)
BMI	34.1 (±8.04)	31.0 (±5.99)
HbA1c	7.2 (±1.40)	7.9 (±1.43)
Ulcer historical duration in weeks	21.5 (5, 392)	16 (5, 104)
Baseline ulcer size (cm^2^)	4.34 (±2.95)	4.43 (±4.70)

In the overall study population included in this analysis (N = 53), the participants were predominantly Caucasian (64%). The majority were male (60%), and 38% were 65 years or older. Fifty-eight percent of participants were obese (BMI ≥ 30), and 18% of participants were overweight (30 > BMI ≥ 25) (Table 1) [22]. There were three outliers for ulcer duration in the dHAICM + SOC group, defined as an ulcer with ≥260 weeks (five years) of historical duration, all of which achieved complete closure with an average time to closure of seven (±3) weeks. Tobacco use and a dorsally located ulcer differed significantly between the dHAICM + SOC and SOC alone groups at baseline; however, due to the limited sample size, statistical significance between the rate of complete closure for dorsal ulcers compared to the rest of the ulcer locations was not calculated. There was no statistically significant difference observed in rates of complete healing between tobacco users and non-tobacco users for either group or the pooled sample (P = 0.65).

Ulcer closure rates

**Figure 1:**
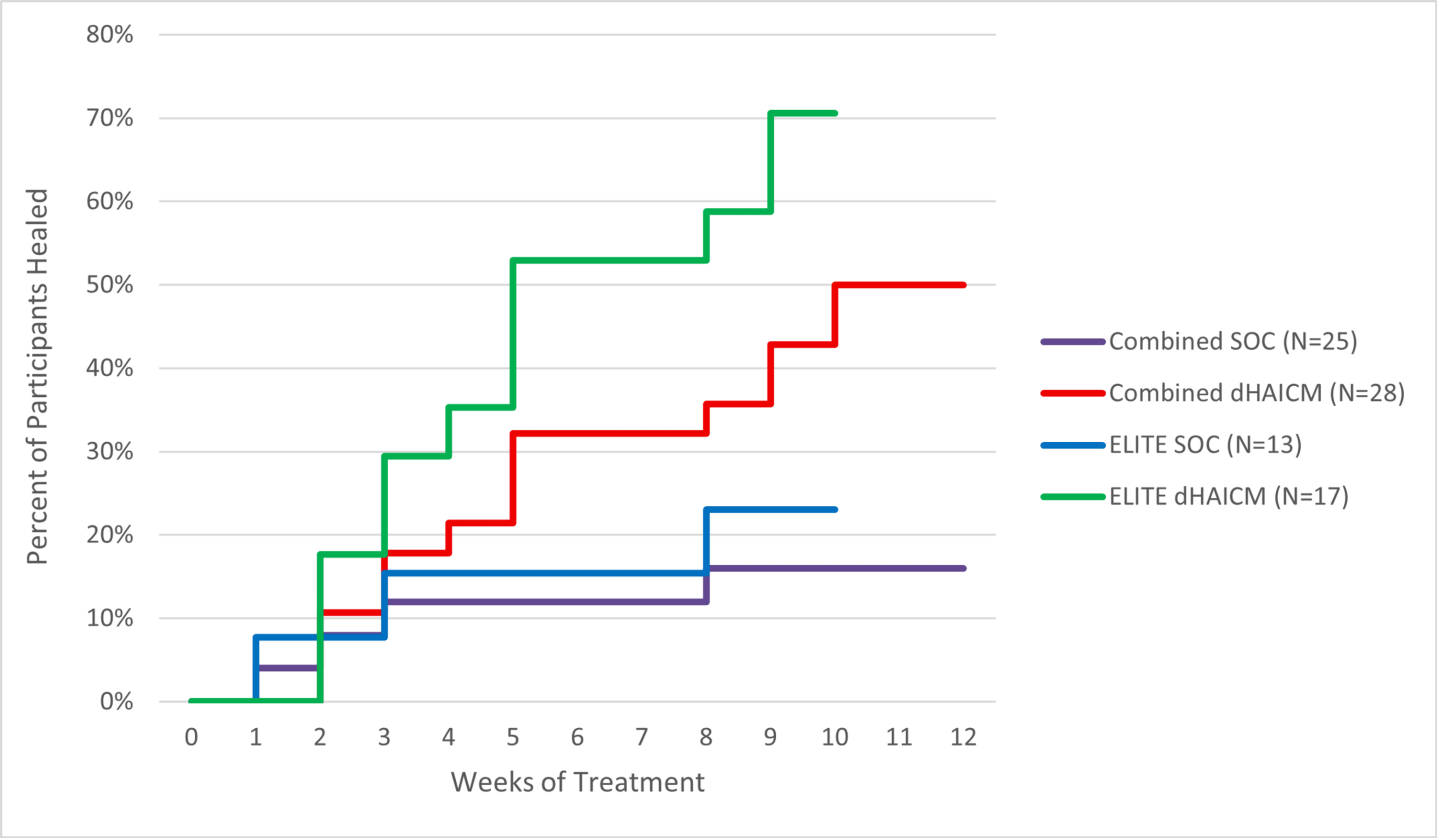
A plot of wound closure rates at each week of treatment by study group No participants achieved wound closure later than week 10 of study treatment. Participants receiving dHAICM + SOC were more likely to heal at all visits beyond week 1 for both the pooled ELITE/CAMPLIFE and ELITE only interim analysis samples. dHAICM: dehydrated human amnion-intermediate layer-chorion membrane; SOC: standard of care

Within the 10-week follow-up period of the ELITE study and the 12-week follow-up period for the CAMPLIFE study, a total of 18 participants (34%) achieved complete wound closure of their ulcers. In the SOC alone group, four participants healed (16%) compared to 14 (50%) in the dHAICM + SOC group (P = 0.011). Utilizing a non-pooled sample from the ELITE study, 12 participants (71%) in the dHAICM + SOC group healed, compared to three participants (23%) in the SOC alone group (P = 0.0253). The risk ratio (RR) for healing in the dHAICM + SOC group was 3.13 for the pooled ELITE/CAMPLIFE analysis and 3.01 for the ELITE only analysis. The percent of participants achieving complete wound closure at each visit is visualized in Figure 1.

**Figure 2:**
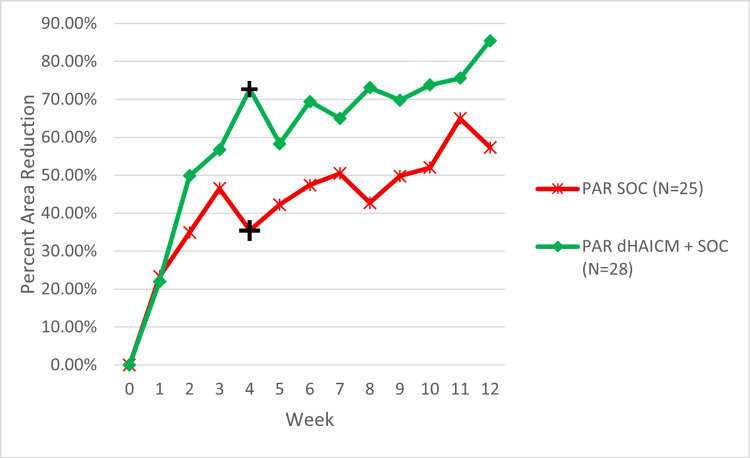
Mean PAR in wound size from baseline each week by treatment group Data represent only the participants who completed a given visit, no data were imputed, so the number of participants represented at each week is lower than the overall N value. ^+^Statistically significant difference P = 0.020, based on unpaired two-tailed Student’s t-test. t = 2.47 (P < 0.05 indicates a statistically significant result). PAR: percent area reduction; dHAICM: dehydrated human amnion-intermediate layer-chorion membrane; SOC: standard of care

For wound area reduction in the pooled ELITE/CAMPLIFE data, a reduction in wound area of 71% ± 18.13% was observed for the dHAICM + SOC group and 46% ± 17.01% for the SOC alone group (Figure 2). However, this reduction did not reach statistical significance (P = 0.0562). In the interim analysis of the data from the ELITE study, a wound area reduction of 78% ± 25.3% was achieved by the dHAICM + SOC vs. a 38% ± 22.4% reduction for SOC alone, a statistically significant difference between the two groups (P = 0.023). All assessed groups had a statistically significant reduction in wound area compared to baseline in both the pooled and ELITE only data samples. The dHAICM + SOC groups, however, exhibited a greater percentage of wound area reduction (Table 2). The PAR data for the pooled analysis are presented in Figure 2.

**Table 2:** Changes in outcome measures from baseline visit to final study visit Quantitative data are presented as mean (±standard deviation). Wound closure data are presented as number (%). N value is lower for some individual data points. P < 0.05 is considered a statistically significant result based on two-tailed Fisher’s exact test for categorical data or two-tailed, unpaired Student’s t-test for quantitative data. *Statistically significant result P = 0.0109 **Statistically significant result P = 0.0253 ***Statistically significant result P = 0.023; t = 2.42 dHAICM: dehydrated human amnion-intermediate layer-chorion membrane; SOC: standard of care; VAS: Visual Analog Scale; WQoL: Wound Quality of Life; FWS: Forgotten Wound Score

	ELITE/CAMPLIFE pooled data		ELITE interim analysis	
Outcome measure	dHAICM + SOC (N = 28)	SOC alone (N = 25)	dHAICM + SOC (N = 17)	SOC alone (N = 13)
Achieved wound closure	14 (50%)*	4 (16%)*	12 (71%)**	3 (23%)**
Achieved wound closure: age ≥ 65 years (dHAICM + SOC N = 11; SOC N = 9)	7 (64%)	2 (22%)	Not evaluated	
Time to closure (weeks)	6 (±3.1)	5 (±4.1)	4.8 (±2.63)	6 (±4.4)
Percent area reduction	71% (±49%)	46% (±43%)	78% (±49%)***	38% (±37%)***
VAS change	-0.06 (±1.64)	-0.2 (±2.14)	0.26 (±1.23)	-0.73 (±2.30)
WQoL change	-0.04 (±0.73)	-0.36 (±0.64)	0.089 (±0.804)	-0.265 (±0.637)
FWS change	12.9 (±33.1)	-0.06 (±32.0)	17.7 (±39.8)	7.28 (±28.0)

Pain and quality of life

Significant differences in pain reduction were not observed between the dHAICM + SOC and SOC alone groups (P = 0.689). Mean VAS pain scores at baseline in the pooled ELITE/CAMPLIFE sample for the dHAICM + SOC group were 1.6, and the SOC alone group was 2.7. No significant reduction was observed in either group for pain scores, with a decrease of 0.06 and a decrease of 0.20 for dHAICM + SOC and SOC alone, respectively. Quality of life assessments showed a similar result, with no significant difference in the changes from baseline between dHAICM + SOC and SOC alone for either the FWS (P = 0.19) or the WQoL (P = 0.114) assessments. Neither group showed a statistically significant change from baseline in FWS with a mean change of 12.9 (P = 0.994) and -0.06 (P = 0.052) for dHAICM + SOC and SOC alone, respectively. For WQoL, the results were similar, with both groups showing an average improvement, with mean reductions of -0.04 and -0.36 for dHAICM + SOC and SOC alone, respectively (P = 0.114). This reduction was statistically significant compared to baseline for SOC alone (P = 0.014), but not for dHAICM + SOC (P = 0.764). A summary of these data is presented in Table 2.

Study completion

The ELITE study had a total of 15 participants who completed the study per protocol due to having fully healed wounds, and three participants completed all treatment visits without attaining a fully healed wound. Five participants withdrew from the study. In the CAMPLIFE study, a total of 14 participants completed the study per protocol, with 11 participants completing all study visits and three participants completing early due to having fully healed ulcers prior to the final treatment visit. Due to administrative closure of the CAMPLIFE study, 11 participants did not complete all assigned follow-up visits and two of those participants did not complete any follow-up visits.

Adverse events

A total of 30 AEs were reported across the two studies. Notably, none of the reported AEs or serious AEs (SAEs) were determined to be related to the studies, procedure, or dHAICM product. The ELITE study had a total of 11 AEs across eight participants, with four of these events meeting the criteria for SAEs. The CAMPLIFE study reported 19 AEs among 12 subjects, with nine of those events meeting the criteria for SAEs.

## Discussion

Chronic DFUs represent a significant pathology with variable outcomes, presenting a difficult challenge for treating clinicians. Compliance with wound maintenance and offloading measures can also vary among patients, further obscuring the effectiveness of treatments. Prospective and controlled evaluations of treatment methods and products are essential to elucidate the comparative effectiveness of treatment paradigms for this significant pathology.

In the present randomized, controlled, multicenter, comparative effective pilot studies, the clinical effectiveness of supplementing the SOC treatment of DFUs with dHAICM compared to SOC treatment alone was evaluated. It was found that supplementing SOC treatments with dHAICM resulted in a greater likelihood of the ulcer healing and a greater reduction in wound area over the treatment period. Although the ulcers did not heal faster in the dHAICM + SOC group compared to the SOC alone group in the pooled ELITE/CAMPLIFE analysis (P = 0.792), the dHAICM + SOC ulcers healed faster in the ELITE only analysis; however, this did not reach statistical significance (P = 0.525). This lack of a statistically significant difference can likely be attributed to the preliminary nature of these data, and we expect the final results of the ELITE study to improve the statistical power of the results.

The overall closure rate over the duration of study treatment (12 or 10 weeks) was utilized to compare the effectiveness of the two treatment modalities. Previously published studies have reported a range of complete healing rates for allograft supplementation to SOC from 45% to 97%, a range from 70% to 97% for human placental tissue allografts specifically, and a range from 8% to 55% for SOC treatment alone [16-19,23]. Our results are in line with this previously published work, with the interim analysis of the ELITE study showing a closure rate of 71% for dHAICM + SOC and 23% for SOC alone, although the closure rate for the pooled CAMPLIFE/ELITE data sample showed a slightly lower closure rate for the dHAICM-supplemented group of 50% and a rate of 16% for SOC alone (Table 2). The differences between the samples could be a result of sample size, inclusion criteria, differences in comorbidities, or patient population characteristics, as sites for the CAMPLIFE study needed to have experience treating both DFUs and venous leg ulcers vs. only DFUs for the ELITE study, potentially leading to differences in patient population characteristics among the two studies. Potentially confounding baseline characteristics, such as ulcer duration or glycemic control (HbA1c), were not statistically significantly different between the dHAICM + SOC and SOC groups at baseline. It is also important to note that in both the CAMPLIFE and ELITE studies, offloading methodologies for plantar ulcers were standardized for all participants and between the treatment and control groups; however, the required methods of offloading varied between the ELITE and CAMPLIFE studies. Additionally, participants required an HbA1c below 12% for enrollment in the ELITE study, a requirement that was not present in the CAMPLIFE study.

PAR of wound area was greater for dHAICM + SOC compared to SOC alone for both the pooled ELITE/CAMPLIFE sample, 71% vs. 46%, and the ELITE interim analysis, 78% vs. 38%; however, this result was only statistically significant for the ELITE sample (P = 0.022). These results align with the published literature that demonstrates wound area reductions ranging from 53% to 98% for allograft supplementation to SOC vs. -2% to 35% for SOC alone, although there is significant variability in the data for wound area reduction in the published literature with reported confidence intervals as large as ±70% of the initial wound area, for SOC treatment, reported by Zelen et al. in their 2013 publication, while the dHPT-supplemented group was notably lower at ±5.8% [16-19,23]. Our data for wound area reduction from baseline to final visit were also subject to variability, with confidence intervals of 71% ± 18.13% for dHAICM + SOC and 46% ± 17.01% for SOC alone. The percentage reduction in wound area was greater for dHAICM + SOC compared to SOC alone at all timepoints beyond week 1, as illustrated in Figure 2; however, this only reached statistical significance at week 4 of treatment (P = 0.02). We attribute this lack of significance in part to the limited sample size at this time at several visits, as well as the variability observed in the data.

Although supplementation with dHAICM did not significantly affect pain reduction (P = 0.679), this can be partially explained by the overall low pain scores at baseline, with a mean VAS of 1.6 for dHAICM + SOC and 2.7 for SOC alone out of 10 at baseline, indicating minimal pain. These findings can likely be attributed to diabetic neuropathy of the foot decreasing the participants’ ability to feel the pain associated with their wounds and, thus, the ability to notice improvements in pain [24]. Further evaluation would be required to validate this hypothesis. Additionally, no significant differences in either the FWS or the WQoL assessments were observed in the dHAICM + SOC group compared to the SOC alone group. There are a number of potential reasons for this lack of difference, despite the greater healing rates for the dHAICM-supplemented group. Many of these participants had multiple ulcers outside of the scope of the study, so improvements in the treated ulcer may have had minimal effect on quality of life. Additionally, since the participants attended weekly visits with mandatory wound dressings, these wounds are unlikely to be forgotten and would likely have a deleterious effect on quality of life, even if they are improving. Finally, many of the participants are used to having multiple chronic wounds over long periods of time and reported minimal pain, so improvements in a wound might have little effect on quality of life.

Important considerations of treatment selection by clinicians for DFUs extend beyond just the relative efficacy of a given treatment. A major impact on care for any patient is the cost-effectiveness and convenience for the clinician to incorporate the treatment into their practice. Although we did not evaluate cost-effectiveness here, dHAICM products are extremely convenient to incorporate into a treatment regimen within a clinical practice. These products are shelf-stable at room temperature for several years and do not require any modifications to SOC for their efficacious use, other than application of the product itself. These qualities lend themselves to the incorporation of dHAICM products into practice for the treatment of DFUs.

Limitations

There are several limitations to the data presented here to consider prior to implementation into practice. Although these data are from randomized controlled studies, the open-label nature means that both investigators and participants were aware of the randomization group upon assignment. DFUs have been estimated to reoccur 60% of the time [7]. Since participants were only observed for two weeks after their wound was assessed as healed, there is the potential for recurrence of the ulcer after the conclusion of participation in the study. For this interim analysis, an independent assessment of wound closure by an independent, medically qualified individual was not performed, relying on the assessment of the unblinded investigator and potentially biasing the assessment. Pooling data from studies with similar but not identical designs could also potentially introduce bias into the data. Additionally, there was a high non-completion rate in the CAMPLIFE study, potentially affecting the quality of the data. We did not account for potential bias in the data resulting from the non-completion of participants in the CAMPLIFE study. We also did not assess the potential effects of the currently enrolled participants in the ELITE study to achieve wound closure prior to the conclusion of their study participation, potentially increasing the percentage of subjects with complete closure in both treatment groups. Finally, as the data from the ELITE study presented here are only from an interim analysis, there is the potential for this analysis to not be representative of the final data.

## Conclusions

Supplementing SOC treatment for DFUs with dHAICM can facilitate a higher rate of complete wound closure, with a higher percentage reduction in wound area, when compared to treatment with SOC alone. Older patients, aged ≥65 years (N = 20), healed more frequently when treated with dHAICM + SOC vs. SOC alone, although this result was not statistically significant (P = 0.09). Increased wound size at baseline (>2.5 cm^2^) increased the likelihood of a wound to heal (P = 0.016) but did not statistically significantly affect the percentage reduction in wound area (P = 0.056) in this analysis. These results are useful in informing clinicians in their treatment of non-healing DFUs; however, they should be interpreted with caution until additional data are available, including final results from the ELITE study. Overall, these data support the use of dHAICM supplementation to SOC for treatment of chronic, non-healing foot ulcers in diabetic patients refractory to initial SOC treatments.
